# Quantile regression application to identify key determinants of malnutrition in five West African countries of Gabon, Gambia, Liberia, Mauritania, and Nigeria

**DOI:** 10.3389/fpubh.2025.1520191

**Published:** 2025-05-26

**Authors:** Reshav Beni, Shaun Ramroop, Faustin Habyarimana

**Affiliations:** School of Mathematics, Statistics and Computer Science, University of KwaZulu-Natal, Pietermaritzburg, South Africa

**Keywords:** health policy, malnutrition, primary healthcare interventions, public health, quantile regression, West African countries

## Abstract

**Background:**

Malnutrition remains one of the most pressing health challenges, particularly in developing nations across Africa, placing a significant burden on both public health agencies and the affected countries. In countries such as Gabon, Gambia, Mauritania, Liberia, and Nigeria, the burden of malnutrition exacerbates public health systems, strains economic resources, and hinders sustainable development, underscoring the urgent need for coordinated efforts at national and international levels. This study aimed to determine the scope and underlying factors contributing to the elevated incidence of malnutrition in West African countries, specifically Gabon, Gambia, Liberia, Mauritania, and Nigeria.

**Method:**

This study employed a quantile regression model to examine the determinants of malnutrition at various quantiles of interest across the Western African countries under consideration to facilitate focused policy measures and intervention strategies aimed at decreasing the prevalence.

**Results:**

For the lower quantiles (0.1 and 0.25), which indicate severe malnutrition, significant variables included the child’s weight [quantile = 0.1, 95% CI(0.0063, 0.0103), quantile = 0.25, 95% CI(0.0054, 0.0107)], mother’s education level [No education: quantile = 0.1, 95% CI(−49.7471, −32.1376), quantile = 0.25, 95% CI(−38.1513, −22.4438) Primary: quantile = 0.1, 95% CI(−24.8095, −5.7693), quantile = 0.25, 95% CI(−19.5273, −6.3424) Higher: quantile = 0.1, 95% CI(5.6499, 40.3274), quantile = 0.25, 95% CI(21.8158, 40.278)], drinking water source [Natural Sources: quantile = 0.1, 95% CI(0.6877, 24.384),Piped: quantile = 0.1, 95% CI(25.578, 45.2368), quantile = 0.25, 95% CI(22.2782, 34.8212), Bottle/Sachet: quantile = 0.25, 95% CI(3.438, 98.1675)], toilet type [Flush: quantile = 0.25, 95% CI(2.2598, 18.3457),Other: quantile = 0.1, 95% CI(8.7863, 24.504), quantile = 0.25, 95% CI(7.0995, 20.1119)], household wealth index [Poorest: quantile = 0.1, 95% CI(−52.5112, −16.9197), quantile = 0.25, 95% CI(−48.3804, −23.0633),Poorer: quantile = 0.1, 95% CI(−38.8744, −4.7586), quantile = 0.25, 95% CI(−34.6993, −9.1766), Middle: quantile = 0.25, 95% CI(−28.9491, −6.5834)], health care visits [No: quantile = 0.1, 95% CI(−19.293, −3.6393), quantile = 0.25, 95% CI(−17.2342, −5.6411)], consumption of fortified foods and tubers [No: quantile = 0.1, 95% CI(−36.3898, −12.0378), quantile = 0.25, 95% CI(−17.8127, −1.2374)], anemia status [Anemic: quantile = 0.1, 95% CI(−15.9326, −1.1929), quantile = 0.25, 95% CI(−12.3361, −1.5516)], mosquito net usage [No: quantile = 0.1, 95% CI(−22.0323, −0.8033), quantile = 0.25, 95% CI(−13.8107, 1.1366)], child’s age [0 to 12 months: quantile = 0.1, 95% CI(81.6424, 105.7155), quantile = 0.25, 95% CI(61.4817, 78.5194),12 to 24 months: quantile = 0.1, 95% CI(0.5592, 24.933), 24 to 36 months: quantile = 0.1, 95% CI(7.9128, 40.2828)] and gender [Female: quantile = 0.1, 95% CI(4.5351, 17.9783), quantile = 0.25, 95% CI(5.0076, 15.4735)], and recent fever [No: quantile = 0.1, 95% CI(11.5663, 29.5984), quantile = 0.25, 95% CI(7.0313, 20.8918)]. Residence type was significant for the 0.25 quantile but not the 0.1 quantile [Rural: quantile = 0.25, 95% CI(−14.7051, −2.1455)]. At higher quantiles (0.75, 0.85, 0.9, and 0.95), factors such as the use of mosquito nets, formula feeding, and access to piped water remain significant, while socioeconomic determinants like maternal education and wealth index lose their influence. Common variables across all quantiles were mother’s age, child’s age (0 to 12 months), child’s gender, and recent fever.

**Conclusion:**

These findings underscore the critical role of primary health care interventions in identifying and managing malnutrition, particularly among lower quantiles where severe malnutrition dominates. High-risk groups, such as teenagers and low-income mothers, should receive targeted support, including prenatal classes and counseling. Community caregivers can monitor at-risk individuals and ensure timely referrals, while collaborations with nonprofits can improve access to food and supplements. Promoting community food gardens, clean water access, and public workshops can further aid prevention and education efforts.

## Introduction

The World Health Organization (WHO) defines malnutrition as “deficiencies or excesses in nutrient intake, imbalance of essential nutrients or impaired nutrient utilization” (1–3). Malnutrition can lead to various dietary-associated non-communicable disorders. In 2016, WHO reported that approximately 155 million children under the age of 5 were stunted, with around 45% of deaths in this age group attributed to undernutrition, predominantly in low- and middle-income countries (1–3). In 2022, 22.3% of children under the age of 5 globally were affected by stunting, a significant decrease from 40.2% in 1990. However, progress has stalled in recent years, and the goal of halving stunting by 2030 remains out of reach (4). In Africa, 30.7% of children under the age of 5 experience stunting, exceeding the global average (5, 6). A similar pattern is evident in the West African countries of interest. In Gabon, 17.0% of children are stunted which is below the African average of 30.7%, whereas Gambia shows a slightly higher rate at 17.5%. Liberia has one of the highest stunting rates globally, with 35.5% of children affected, followed by Mauritania at 25.1% and Nigeria at 40% (5–9).

Anthropometric parameters, including mid-upper arm circumference (MUAC), weight, height, and age, are essential in categorizing different forms of undernutrition. Wasting, characterized by a low weight-for-height z-score (WHZ), often indicates acute, severe weight loss typically associated with food insecurity and acute illness. Stunting, identified by a low height-for-age z-score, reflects chronic or recurring undernutrition, often linked to factors such as poverty, poor maternal health, frequent or chronic diseases, low birth weight, and neonatal factors. Underweight is determined by a low weight-for-age z-score, and an underweight child may also be stunted or wasted. Additionally, micronutrient deficiencies, which affect around 2 billion people globally, result from inadequate intake of essential vitamins and minerals necessary for optimal growth and development. These deficiencies often involve multiple micronutrients, such as vitamin A, folate, iron, iodine, and zinc, and may arise from maternal undernutrition during pregnancy and lactation or socioeconomic challenges related to poverty and food insecurity (10).

Undernutrition severity is classified as moderate acute malnutrition (MAM) or severe acute malnutrition (SAM), with MUAC measurements used for diagnosis. MAM is indicated by a MUAC between 11.5 cm and < 12.5 cm, while SAM is diagnosed if a child has a MUAC of <11.5 cm or presents with nutritional edema (11). SAM diagnosis is associated with a mortality rate 10 times higher than that of children with a z score ≥ −1 (12).

Statistical models with the regression method have traditionally been employed to analyze childhood malnutrition across sub-Saharan African countries. However, previous approaches have primarily focused on mean regression rather than quantile regression (13). Utilizing quantile regression offers greater suitability in modeling malnutrition, as evidenced by extensive literature examples in Shibeshi and Asfaw (14), Seboka et al. (15), and Mtambo et al. (16). This method allows for the analysis of determinants corresponding to various quantiles of interest, such as the lower tail (e.g., 5% or 10%), upper tail (e.g., 90% or 95%), or median (50%) of the distribution, as opposed to solely analyzing the determinants of the mean distribution (16).The primary objective of this study (and by implication the hypothesis) is to employ a quantile regression model to examine the determinants of malnutrition at various quantiles of interest across the Western African countries under consideration include Gabon, Gambia, Liberia, Mauritania, and Nigeria to facilitate focused policy measures and intervention strategies aimed at decreasing the prevalence.

The present study represents a significant contribution in the field by introducing a novel framework utilizing quantile regression analysis to explore the determinants of malnutrition, while also incorporating dietary-related variables. Notably, there exists a dearth of literature that employs quantile regression techniques on datasets spanning multiple West African countries to identify the pivotal factors influencing malnutrition. This research addresses this gap, underscoring its innovative approach and potential to advance the understanding of malnutrition dynamics within Western African countries.

## Methodology

Quantile regression was first introduced by Koenker and Basset (17). This regression technique is a statistical method used to model conditional quantiles of an outcome variable in relation to covariates, rather than focusing solely on the mean. This approach provides a robust framework for analyzing non-normal data and addressing statistical outliers. Compared to mean regression, quantile regression offers greater flexibility and insight into the underlying associations, especially when modeling anthropometric measurements and nutritional status. By analyzing quantiles at different percentiles of the distribution, such as the lower tail, median, or upper tail, quantile regression allows for a comprehensive examination of the determinants of nutritional status across various levels of the population distribution. This approach has been extensively supported in the literature as a more appropriate method for capturing the complexities of nutritional data and providing valuable insights into health determinants (13).

Let Y be the target variable of interest and X a vector of observed covariates. The quantile, denoted by, of Y conditional on X=x can be modelled using the quantile regression model expressed as:


$$Q_{y_{i}|x_{i}}(\tau |x_{i})=x_{i}^{T}\beta_{\tau }$$

Where, $Q_{y_{i}|x_{i}}(\tau |x_{i})$ is the condition $\tau^{\mathrm{th}}$ quantile outcome given $x_{i_{\tau }}$ τ∈(0,1) is the $\tau^{\mathrm{th}}$ quantile of the outcome variable (13).

The quantile regression model, once fitted, can be represented mathematically using the following equation:


$$Q_{y_{i}|x_{i}}=b_{0\tau }+b_{1\tau }x_{1i}+\ldots +b_{\mathrm{k\tau}}x_{\mathrm{ki}}+\varepsilon_{\mathrm{i\tau}},i=1,2,\ldots ,n.$$

Where, $Q_{y_{i}|x_{i}}$ represents the quantile of the outcome variable *y*; τ denotes the quantile level; $b_{0\tau },b_{1\tau },\ldots ,b_{\mathrm{n\tau}}$ denotes the coefficients per an independent variable at a specified quantile τ ; $\varepsilon_{\mathrm{i\tau}}$ denotes an error term; $x_{1i},x_{2i},\ldots ,x_{\mathrm{ki}}$ represent the independent variables.

### Data

Demographic and Health Surveys (DHS) conducted within West African countries served as the primary data source for this secondary data analysis. The DHS employs a complex, multistage sampling design to ensure that the survey produces nationally representative estimates.

Specifically, the sampling design involves two-stage stratified cluster sampling. In the first stage, primary sampling units, typically corresponding to census enumeration areas, are selected based on probability proportional to size. Stratification is performed before sampling by dividing the population into subgroups, such as urban and rural areas within each region, to improve the precision of the survey estimates.

In the second stage, within each selected primary sampling unit, a systematic sample of households is drawn. This two-stage procedure ensures that all households have a known and non-zero probability of selection, though not necessarily equal, leading to the need for the application of sampling weights. These weights adjust for differences in the probability of selection and for non-response, allowing for unbiased, nationally representative estimates.

Given the complex design, design effects and clustering must be accounted for in all statistical analyses to ensure correct estimation of standard errors, confidence intervals, and significance tests. In this study, appropriate survey weights, stratification, and clustering variables, as provided by DHS, were incorporated into all analyses in accordance with DHS analytical guidelines.

**Figure 1:**
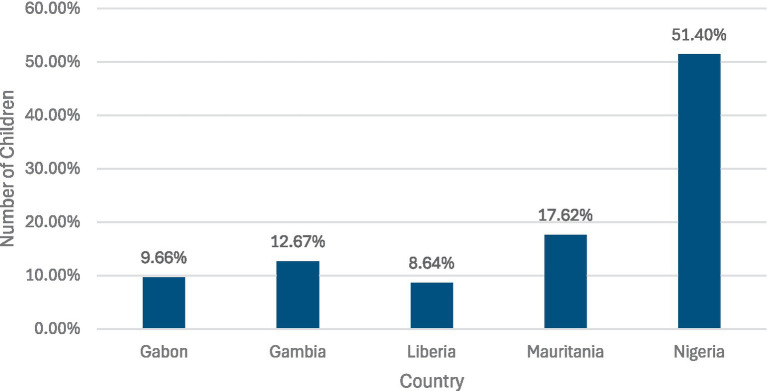
Population distribution of the dataset.

Under the guidance of the DHS manual, the data used were extracted from the Kids Recode table, which has a unit of analysis of a child under the age of 5 born to a woman interviewed. A dataset was created by stacking these various datasets for the following countries: Gabon (2019–2021), Gambia (2019–2020), Liberia (2019–2020), Mauritania (2019–2021), and Nigeria (2021). Missing data was removed from the dataset. The dataset contained a total of 65,994 observations, with the distribution of the pooled data source being outlined in Figure 1.

The dependent variable of interest was the height-for-age z score (HAZ). According to World Health Organization (1–3), a child is considered malnourished when HAZ was less than −2.0 and was considered nourished when the HAZ was greater than −2.0.

This current scientific setting examined demographic, socioeconomic and environmental factors related to child malnutrition that have been explored in numerous prior studies. These factors served as the foundation for the present study, which led to the following potential independent variables being identified: region of the respondent, current age of the mother, type of residence, highest level of education obtained by the mother, source of drinking water, type of toilet facility, whether the household has electricity or not, gender of the head of the household, age of the head of the household, wealth of the household, whether the respondent slept under a mosquito net last night, whether the respondent visited a health facility in the last 12 months, whether the child was given milk or not, whether the child was given formula or not, whether the child was given fortified food or not, whether the child was given potatoes, cassava, or other tubers or not, whether the child was given eggs or not, whether the child was given meat or not, anemia level, whether the household has a mosquito bed net for sleeping or not, age of the child, gender of the child, whether the child had a fever in the last 2 weeks or not, whether the child had a cough in the last 2 weeks or not.

The theoretical framework employed was grounded in existing literature (13, 18–21). The framework employed draws on established theories and concepts to guide the selection of variables that are hypothesized to influence malnutrition.

### Descriptive analysis

The descriptive statistics presented in the Table 1 provide a comprehensive overview of the study population’s key variables, highlighting both continuous and categorical data. The target variable, height-for-age standard deviation, reflects an average of −106.21 with a wide range (−598 to 598), indicating notable variability in child growth outcomes. Mothers in the sample are, on average, 29.62 years old, while household heads average 42.92 years, with ages ranging broadly from 14 to 98. Socioeconomic indicators reveal significant disparities: 41.27% of mothers have no formal education, and only 5.37% have attained higher education. Most households are rural (59.90%) and rely on wells (47.29%) or piped water (30.76%) as drinking water sources. Sanitation varies, with 46.04% using pit latrines and only 24.31% having access to flush toilets. Wealth distribution skews toward the lower end, with 27.85% of households classified as “poorest” and only 12.46% as “richest.” Nutritional and health behaviors underscore challenges in child nutrition and care. While 60.87% of respondents visited a health facility in the past year, the dietary data show that less than 20% of children were given milk, fortified food, or meat, and 80.58% are classified as anemic. Additionally, 67.83% of households own mosquito nets, but 51.98% of children did not sleep under any net the previous night. Child demographics reveal an even gender distribution and balanced representation across age groups, with around 20% of children in each age category from 0 to 60 months. While 22.42% of children experienced fever in the last 2 weeks, 17.36% reported a cough, pointing to recurring health concerns in the population. These findings highlight the complex interplay of demographic, socioeconomic, and environmental factors that influence health outcomes in this context.

**Table 1:** Descriptive statistics of the participants.

Variable name	Categories	% or Average or Range
Height/age standard deviation (target)	Continuous	Avg = −106.21(min: −598, max: 598)
Mothers age	Continuous	Avg = 29.62 (min: 15, max: 49)
Age of the head of the household	Continuous	Avg = 42.92 (min: 14, max: 98)
Child weight (kg)	Continuous	Avg = 894.87 (min: 221, max: 9996)
Mothers highest level of education	No education	41.27%
	Primary	22.71%
	Secondary	30.65%
	Higher	5.37%
Residence type	Rural	59.90%
	Urban	40.10%
Type of water drinking source	Bottle/sachet	0.44%
	Natural sources	13.18%
	Tanker	3.20%
	Piped	30.76%
	Well	47.29%
	Other	5.14%
Type of toilet facility	Flush	24.31%
	Pit	46.04%
	Other	29.65%
Does the household have electricity	No	0.5215
	Yes	0.4785
Gender of the head of the household	Female	0.191
	Male	0.809
Wealth index	Poorest	27.85%
	Poorer	22.69%
	Middle	20.59%
	Richer	16.41%
	Richest	12.46%
Type of mosquito bed net(s) slept under last night	No net	51.98%
	Only treated nets	44.92%
	Both treated and untreated	0.01%
	Only untreated nets	3.08%
Visited health facility last 12 months	No	39.13%
	Yes	60.87%
Gave child tinned, powdered or fresh milk	No	81.73%
	Yes	18.27%
Gave child baby formula	No	90.93%
	Yes	9.07%
Gave child fortified baby food (cerelac, etc.)	No	92.65%
	Yes	7.35%
Gave child potatoes, cassava, or other tubers	No	82.02%
	Yes	17.98%
Gave child eggs	No	90.36%
	Yes	9.64%
Gave child meat (beef, pork, lamb, chicken, etc.)	No	87.40%
	Yes	12.60%
Whether the child is Anemic or not	Anemic	80.58%
	Not Anemic	19.42%
Have mosquito bed net for sleeping	No	32.17%
	Yes	67.83%
Current age of child (months)	0 to 12 months	21.30%
	12 to 24 months	19.77%
	24 to 36 months	19.17%
	36 to 48 months	19.70%
	48 to 60 months	20.05%
Gender of the child	Female	49.02%
	Male	50.98%
Had fever in last 2 weeks	No	77.58%
	Yes	22.42%
Had cough in last 2 weeks	No	82.64%
	Yes	17.36%

## Results

Quantile regression offers a comprehensive and robust approach to analyzing the determinants of malnutrition, particularly in the context of Western African nations like Gabon, Gambia, Liberia, Mauritania, and Niger. By examining multiple quantiles, quantile regression provides a flexible and interpretable framework for capturing heterogeneity in the data, offering insights into how the effects of covariates vary across different segments of the outcome variable’s distribution (17, 22). Therefore, incorporating granular conditional quantiles in the analysis enhances the understanding of the factors influencing malnutrition and provides valuable insights for targeted intervention strategies.

A quantile regression model was applied to the sourced DHS data using a SAS QUANTREG procedure from SAS Enterprise Guide version 8.1, the results of which are discussed below. Supplementary Table 1 contains a summary of the results obtained from the various levels of quantiles explored (0.1, 0.25, 0.5, 0.75, 0.85, 0.9, and 0.95). A significance level of 0.05 was used. The quantile levels selected are aligned to research done by Abdulla et al. (23) and MokallaI and Mendu (24).

The analysis reveals that a mother’s current age is a significant determinant of malnutrition across all quantiles, with increasing importance from quantile 0.25 onwards. The child’s weight is significant only for quantiles 0.1, 0.2, and 0.5, where the coefficients remain relatively stable. The type of residence, using “Urban” as the reference, is only significant for the 0.25 quantile. The mother’s education level, with “Secondary” as the reference, shows that all education levels are significant from quantile 0.1 to 0.85, except for “No education” at quantile 0.90, and no levels are significant at quantile 0.95. The source of drinking water, with “Well” as the reference, indicates that the “Bottle/Sachet” category is significant at quantiles 0.25 and 0.5, “Natural sources” at quantiles 0.1, 0.5, 0.75, and 0.85, and “Piped” from quantile 0.1 to 0.9, while “Other” is significant only at the 0.5 quantile.

Regarding toilet facilities, with “Pit” as the reference, “Flush” is significant for all quantiles except 0.1, and “Other” is significant across most quantiles except 0.95. The gender of the household head, using “Male” as the reference, is significant only at the 0.5 quantile. The wealth index, with “Richest” as the reference, shows that the “Poorest” category is significant across all quantiles except 0.9 and 0.95, while “Poorer” is significant similarly but also insignificant at 0.85. The “Middle” category is significant only at quantiles 0.25, 0.5, and 0.75, and “Richer” is not significant in any quantile.

The use of mosquito nets, with “Only untreated nets” as the reference, is significant at quantiles 0.5, 0.75, 0.85, and 0.90 for “No net” and at quantiles 0.5 and above for “Only treated nets.” Whether the child visited a healthcare facility in the last 12 months, with “Yes” as the reference, is significant in all quantiles except 0.75 and 0.85. Formula feeding, with “Yes” as the reference, is significant at quantiles 0.5, 0.75, and 0.90. Receiving fortified foods, with “Yes” as the reference, is significant only at quantiles 0.1 and 0.25, while giving tubers is significant at quantiles 0.1, 0.25, and 0.5, and giving eggs is significant at quantiles 0.5 and 0.75. Giving meat is significant across all quantiles except 0.1 and 0.25.

Anemia presence, with “Not Anemic” as the reference, is significant only at quantiles 0.1 and 0.25. The presence of a mosquito net, with “Yes” as the reference, is significant only at quantiles 0.1 and 0.95. The age of the child, with “48 to 60 months” as the reference, shows that “0 to 12 months” is significant across all quantiles, “12 to 24 months” is significant for all but quantiles 0.25 and 0.95, “24 to 36 months” is significant only at quantiles 0.1 and 0.95, and “36 to 48 months” is not significant in any quantile. Lastly, whether the child had a fever in the last 2 weeks, with “Yes” as the reference, and the gender of the child, with “Male” as the reference, are significant across various quantiles, while whether the child had a cough in the last 2 weeks is significant only at quantiles 0.5 and 0.95. Variables like household electricity and giving milk showed no significant relationship with malnutrition across the quantiles.

Annexure A provides a concise overview of the quantile regression results, focusing on significant variables. The graphs display individual coefficients with a 95% confidence interval. Within Annexure A, the intercept represents the estimated conditional quantile function for malnutrition in children under 5 years old, showing a less negative impact in the upper quantiles compared to the lower ones.

For the Residence type variable, a declining trend is seen for quantiles below 0.25, followed by an upward trend up to quantile 0.5, and a slight decline above 0.5. The mother’s current age variable shows a decreasing slope below quantile 0.25 and an increasing trend above it, indicating a greater positive impact in the upper quantiles. Children of mothers with no education exhibit an increasing trend across quantiles, with more severe impacts at lower quantiles, unlike mothers with primary or higher education, who show a declining trend around quantiles 0.25 and 0.5.

Households with piped drinking water sources display a decreasing positive impact across quantiles, while those with natural sources, sachet/bottle, tanker, and other sources show less clear trends with some reversals. A rising positive trend is noted for households with a flush toilet facility up to quantile 0.75, followed by a slight decline, whereas households with other toilet facilities show a decreasing trend up to quantile 0.5, followed by an increase.

The age of the head of the household generally shows a decreasing trend across quantiles. The wealth index reveals an increasing trend for “Poorer” and “Poorest” households, with a trend break for the “Poorest” from quantile 0.75 onwards. “Middle” and “Richer” households initially show a decreasing trend, with a reversal at quantiles 0.50 and 0.85.

For children who did not sleep under a mosquito net, there is a declining trend up to quantile 0.25, continuing steadily downward, with a similar trend for those who slept under a treated net. Children who did not visit a healthcare facility in the last 12 months show an inclining trend across all quantiles. A declining trend is seen for children who did not consume baby formula, with an increasing trend between quantiles 0.75 and 0.85. Children who did not receive fortified baby foods and tubers show an increasing trend up to quantile 0.75, followed by a decline and an uptick at quantile 0.90, while those who received eggs show a declining trend up to 0.75, then an increase.

Children who were anemic and those who did not use a mosquito net both exhibits increasing trends, moving from negative to positive coefficients at quantiles 0.65 and 0.55, respectively. The age of the child shows a decreasing trend up to quantile 0.9, where it shifts to an increasing trend. For female children, a positively increasing trend is observed. Children without a fever or cough in the last week initially show a decreasing trend up to quantiles 0.25 and 0.5, followed by an increasing trend. Lastly, no significant trend is observed for the child’s weight variable.

## Discussion

Malnutrition remains a significant challenge in Western Africa contributing substantially to pediatric morbidity and mortality rates (25). Despite efforts by Western African governments, non-profit organizations, and humanitarian initiatives to mitigate its effects through healthcare policies and enhanced access to care, a more proactive response is necessary to address this health crisis effectively.

This study draws upon data from the DHS. Its primary aim is to discern the factors influencing malnutrition in children aged 59 months or younger. By identifying at-risk populations promptly and implementing preventive measures, the study seeks to enhance pediatric health outcomes.

When referring to the lower quantiles, namely 0.1 and 0.25, which relate to the most severe level of malnutrition, the common variables that are deemed to be significant for both of the lower quantiles were the weight of the child, the highest level of education obtained by the mother, the source of drinking water, type of toilet facility, the wealth index of the household, whether the child visited a health care facility in the last 12 months or not, whether the child was given fortified foods, whether the child was given tubers, whether the child was anemic or not, whether the child slept under a mosquito last night or not, the age of the child, the gender of the child and whether the child had a fever in the last 2 weeks or not. It should be noted that the residence type was deemed to be significant for the 0.25 quantile and not the 0.1 quantile.

Furthermore, there are common variables that were observed to be significant regardless of the quantile in which was under analysis, these variables were the current age of the mother, the age of the child the “0 to 12 months” category, the gender of the child and whether a fever was present in the last 2 weeks or not.

The findings of this study are comparable to other papers in literature such as a paper by Sharaf et al. (20) who applied a quantile regression technique to 2014 Egypt Demographic and Health Survey data to identify key risk factors of malnutrition using HAZ as a target variable; the study noted that the level of the mothers education, mother age and the wealth of the household are significant determinants of malnutrition. A Bayesian quantile regression approach was applied by Gayawan et al. (26) on 2013 Nigeria Demographic and Health Survey data which noted that the level of a mothers education, household wealth, type of toilet facility, whether a child had a fever in the last 2 weeks or not are all significant risk factors in relation to malnutrition. Furthermore, the study also found that whether a child had a cough in the last 2 weeks and whether the household had electricity or not was not a significant factor in determining malnutrition. However, Gayawan et al. (26) did find contrary results to this paper where the source of drinking water was deemed to be an insignificant determinant of malnutrition. A study by Aheto (13) employed a multivariable simultaneous quantile regression model to identify determinants for severe stunting based on HAZ on data from the 2014 Ghana Demographic and Health Survey; identified gender, age of the child, mothers age, mothers educational level and wealth index as significant contributors to malnutrition as well. However, the study did not find the variables that denotes whether a child had a fever in the last 2 weeks to be a significant risk factor for malnutrition which is contrary to the findings within this paper. Shiratori (21) utilized a quantile regression approach onto data from 2010 Tanzania Demographic and Health Survey to identify the key determinants of malnutrition using HAZ as an indicator of malnutrition; the study showed that the level of the mother’s education, the wealth index of the household, gender of the child and type of toilet facility are significant risk factors of malnutrition. However, in contrast to the findings of this paper, Shiratori (21) noted that the type of residence of the child is not a significant determinant of malnutrition.

These findings underscore the necessity for further investigation into the factors influencing malnutrition in Sub-Saharan Africa, with a specific emphasis on Gabon, Gambia, Liberia, Mauritania, and Nigeria. The findings highlight the importance of targeted primary healthcare interventions, especially for high-risk pregnancies in low-income households. Identifying and supporting young women with low education and wealth levels is crucial. Providing access to contraception, prenatal education, and continuous monitoring by community health workers can help prevent and manage malnutrition. During postnatal visits, regular measurements and prompt referrals for malnourished children are essential to ensure appropriate care (27, 28).

Local governments should collaborate with nonprofits to provide food aid, fortified foods, and high-protein supplements to families of malnourished children. Community food gardens and clean water access should be prioritized to empower local women and improve child nutrition. Government infrastructure programs should address water and sanitation needs in areas with high malnutrition rates. Providing mosquito nets to children at birth and organizing regular community outreach initiatives can further mitigate malnutrition risks. Technology offers potential solutions, such as enrolling high-risk mothers in webinars on antenatal care or utilizing offline mobile health apps for prenatal and antenatal guidance (31, 32).

Future research endeavors may extend the scope of variables examined, especially financial metrics, by incorporating gross domestic product as a variable of interest. Additionally, a comprehensive exploration of health determinants could encompass chronic ailments and infectious diseases, including prior or current tuberculosis infection, prevalence of human immunodeficiency virus (HIV), and HIV disease stage. Moreover, the impact of prenatal factors such as gestational age and prenatal vitamin supplementation, as well as postnatal factors like low birth weight, feeding practices, vaccination rates, vitamin A supplementation, deworming, and postnatal healthcare visits, on nutritional outcomes warrants investigation. Social factors such as primary caregivers and eligibility for government subsidies could also be scrutinized. Employing longitudinal analysis methods may unveil significant trends over time. Furthermore, the inclusion of spatial variables or more granular quantiles could also be valuable. Alternative methodologies, such as generalized additive models or spatial quantile regression, can further be employed.

## Conclusion

This study employed a quantile regression model to investigate the determinants of childhood malnutrition between 2019 and 2021 across five West African countries: Gabon, Gambia, Liberia, Mauritania, and Nigeria. The findings highlight a range of factors significantly associated with malnutrition, particularly among children situated in the lower tails of the height-for-age z-score distribution, where severe malnutrition is most pronounced. Key determinants included child weight, maternal education level, household wealth, access to clean drinking water and sanitation facilities, dietary diversity, anemia status, healthcare access, use of mosquito nets, and the child’s age and gender. These results emphasize the critical need for multi-sectoral interventions targeting both socioeconomic and health-related risk factors to effectively reduce malnutrition and improve child health outcomes within these regions.

### Limitations

The study’s limitation lies in its reliance on cross-sectional data, suggesting that longitudinal research could uncover more significant patterns.

### Recommendations

Based on the study’s findings, several key recommendations are proposed to policymakers, health practitioners, and researchers. Targeted interventions are urgently needed to support high-risk groups, particularly young mothers with limited education and low socioeconomic status. Strategies should include improving access to prenatal and postnatal healthcare services, enhancing community-based nutrition education, and providing fortified foods and micronutrient supplements. Investments in infrastructure to ensure safe drinking water and proper sanitation, alongside distribution of mosquito nets and malaria prevention initiatives, are also critical. Collaborative efforts between governments, non-profit organizations, and community groups can foster sustainable food security initiatives, such as local food gardens and nutrition support programs. Future research should prioritize longitudinal studies to capture temporal patterns of malnutrition and explore the inclusion of more detailed socioeconomic and health-related variables to enrich the understanding of malnutrition dynamics in West Africa.

## Data Availability

The data analyzed in this study is subject to the following licenses/restrictions: the data is available upon request from DHS program. Requests to access these datasets should be directed to www.dhsprogram.com.
