# Supplementary material for: Quantile regression application to identify key determinants of malnutrition in five West African countries of Gabon, Gambia, Liberia, Mauritania, and Nigeria
Source: Front Public Health. 2025 May 26;13:1520191. doi: 10.3389/fpubh.2025.1520191 (PMC12146170; doi:10.3389/fpubh.2025.1520191)
Supplement: Supplementary file 1 [file Supplementary_file_1.docx]

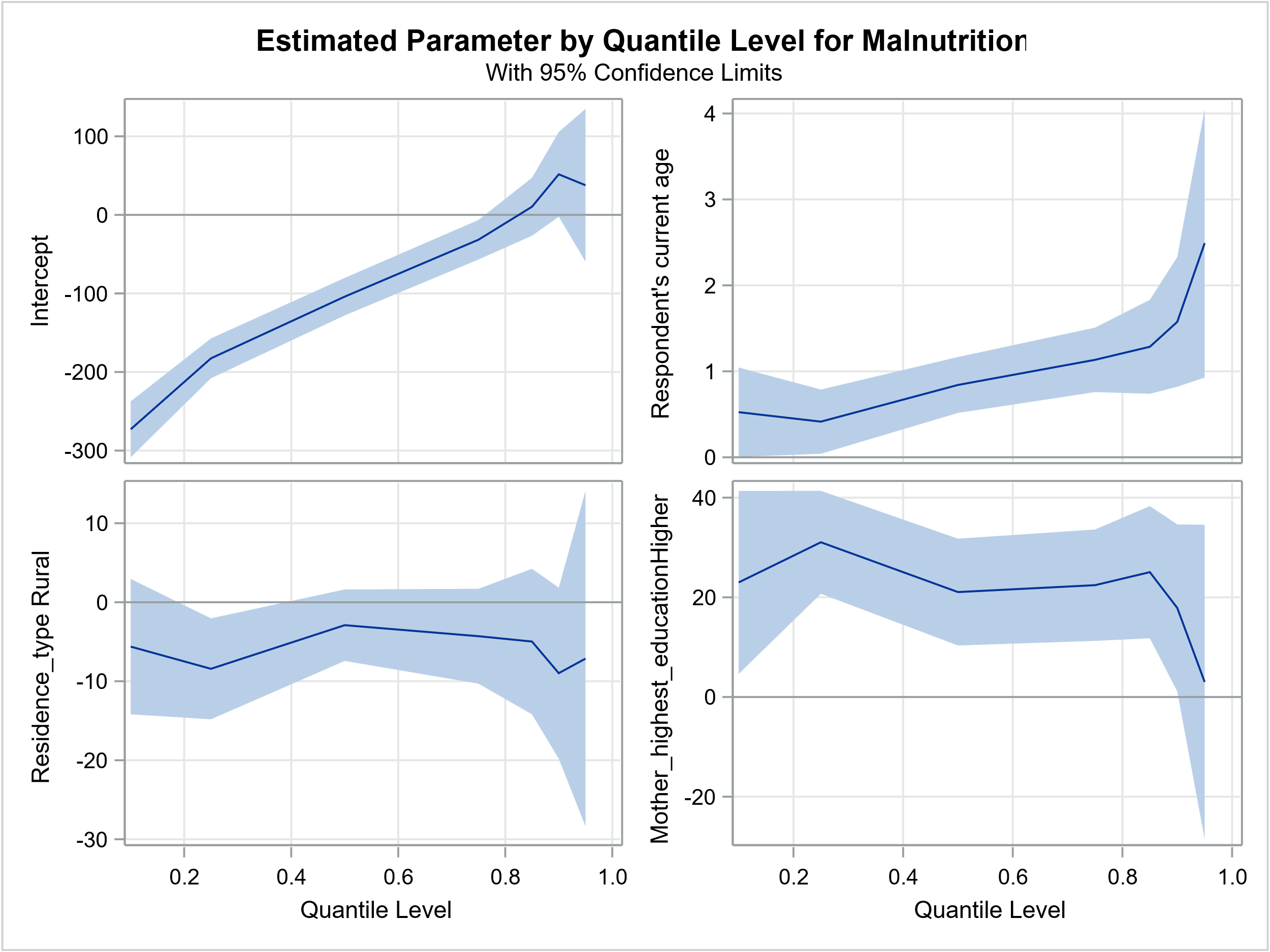
Annexure A


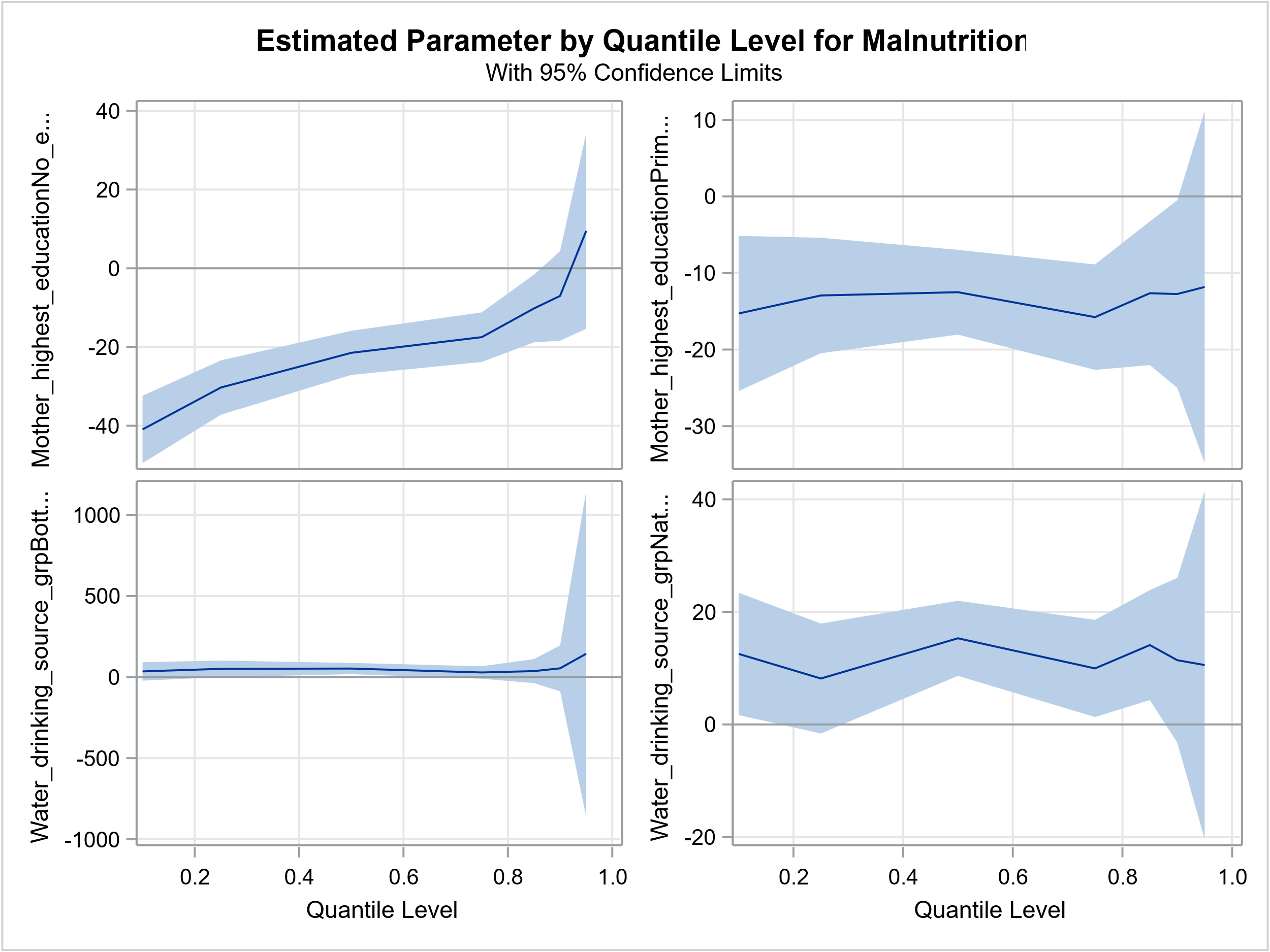


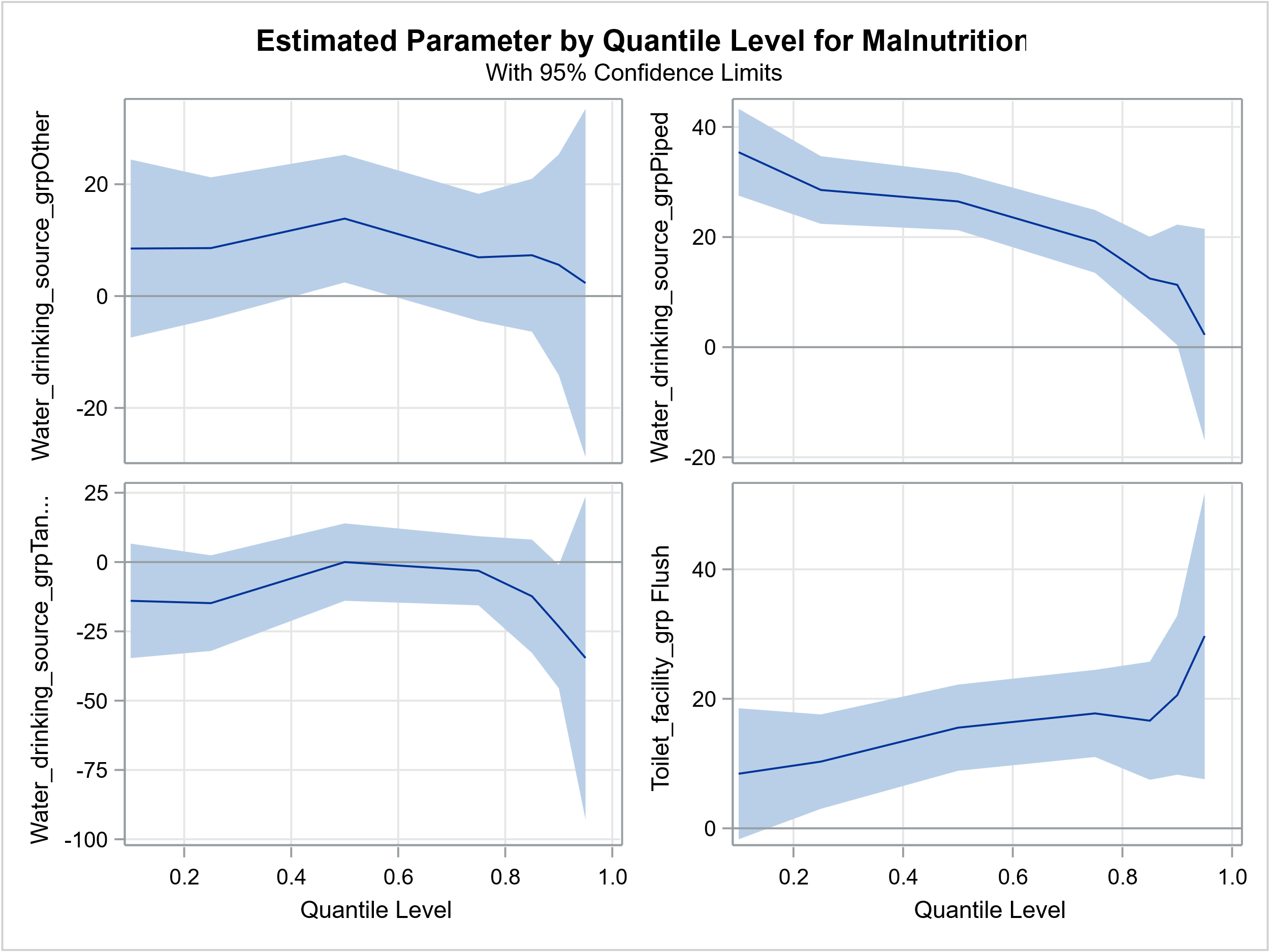


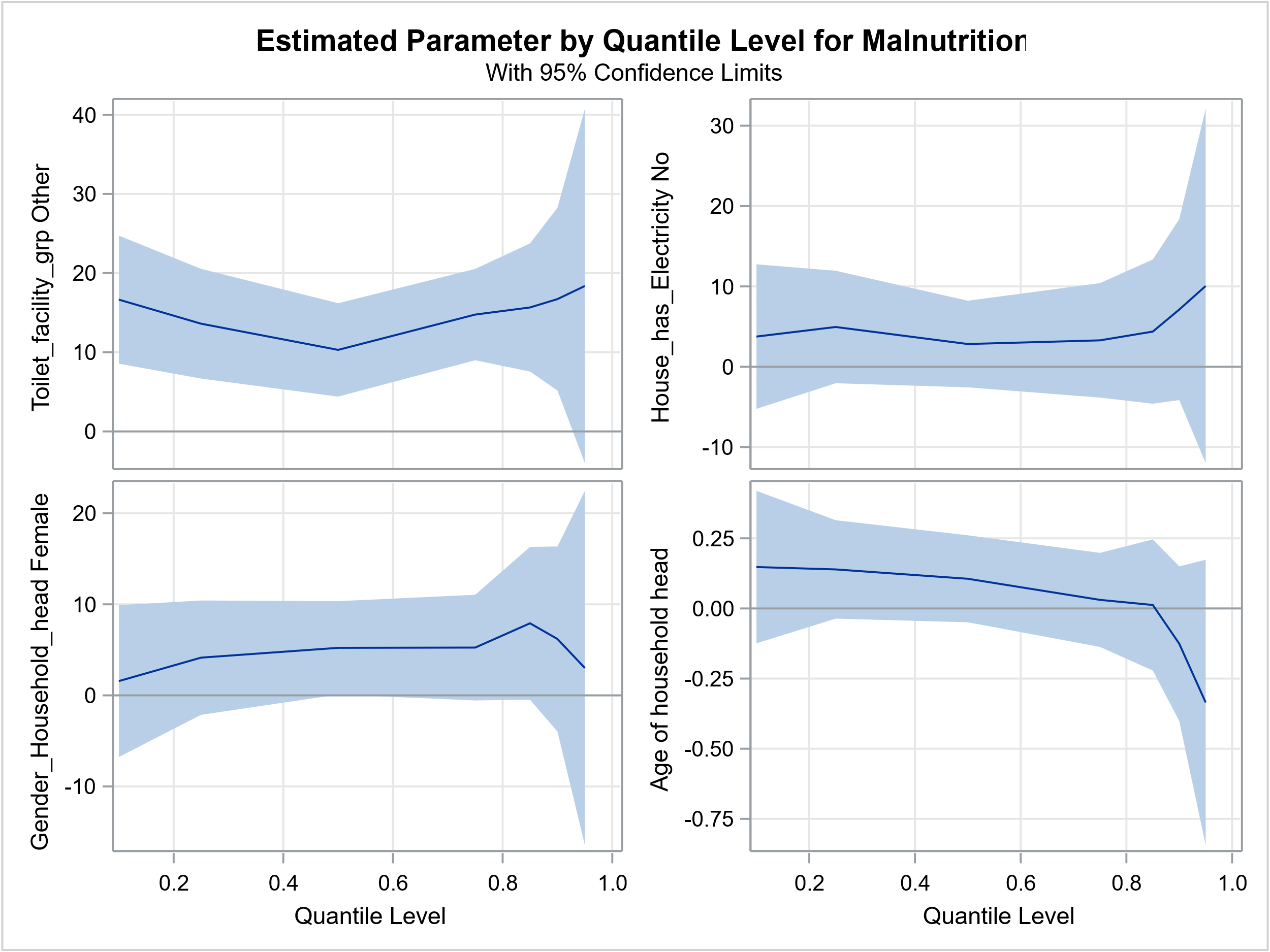


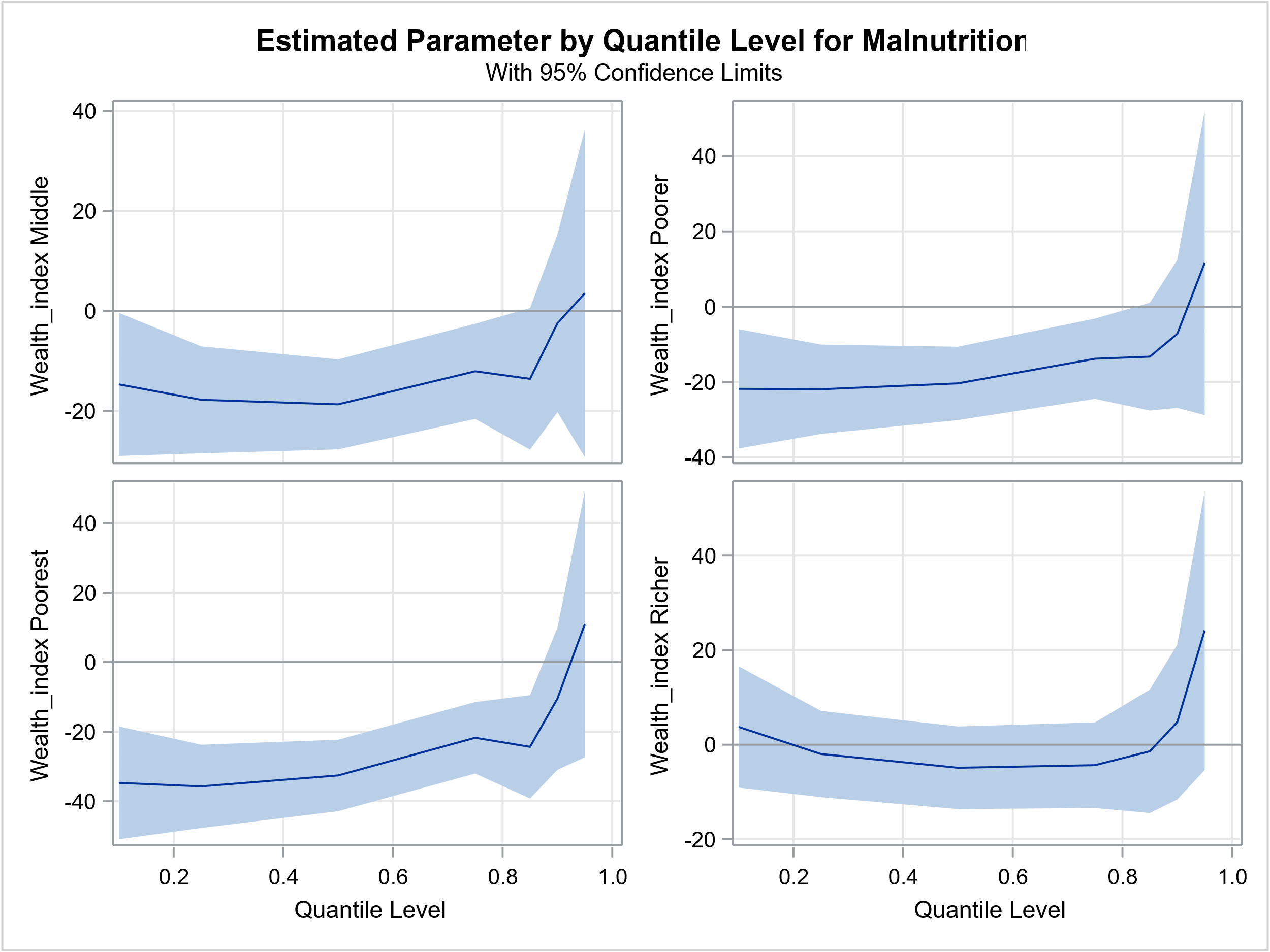


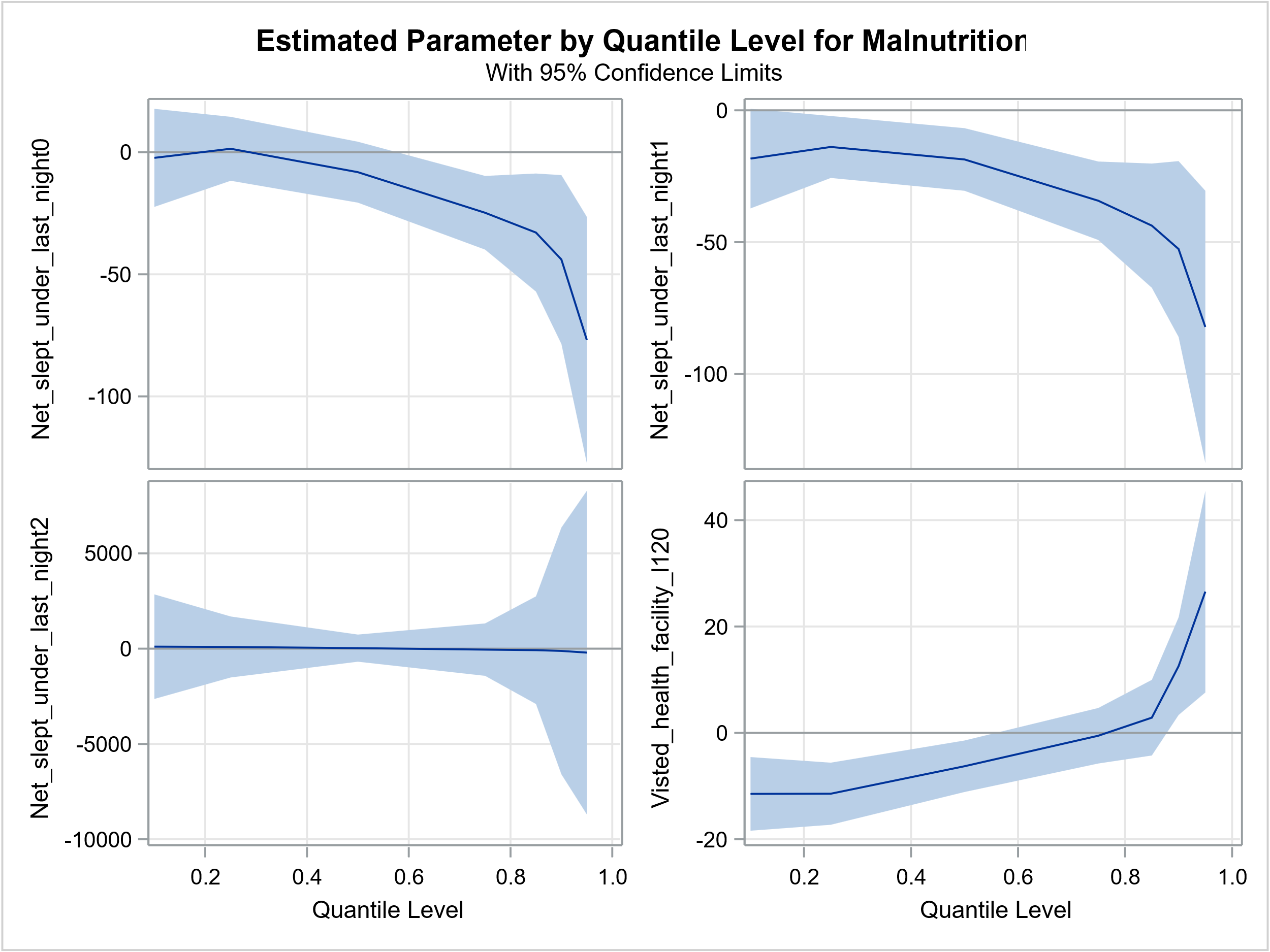


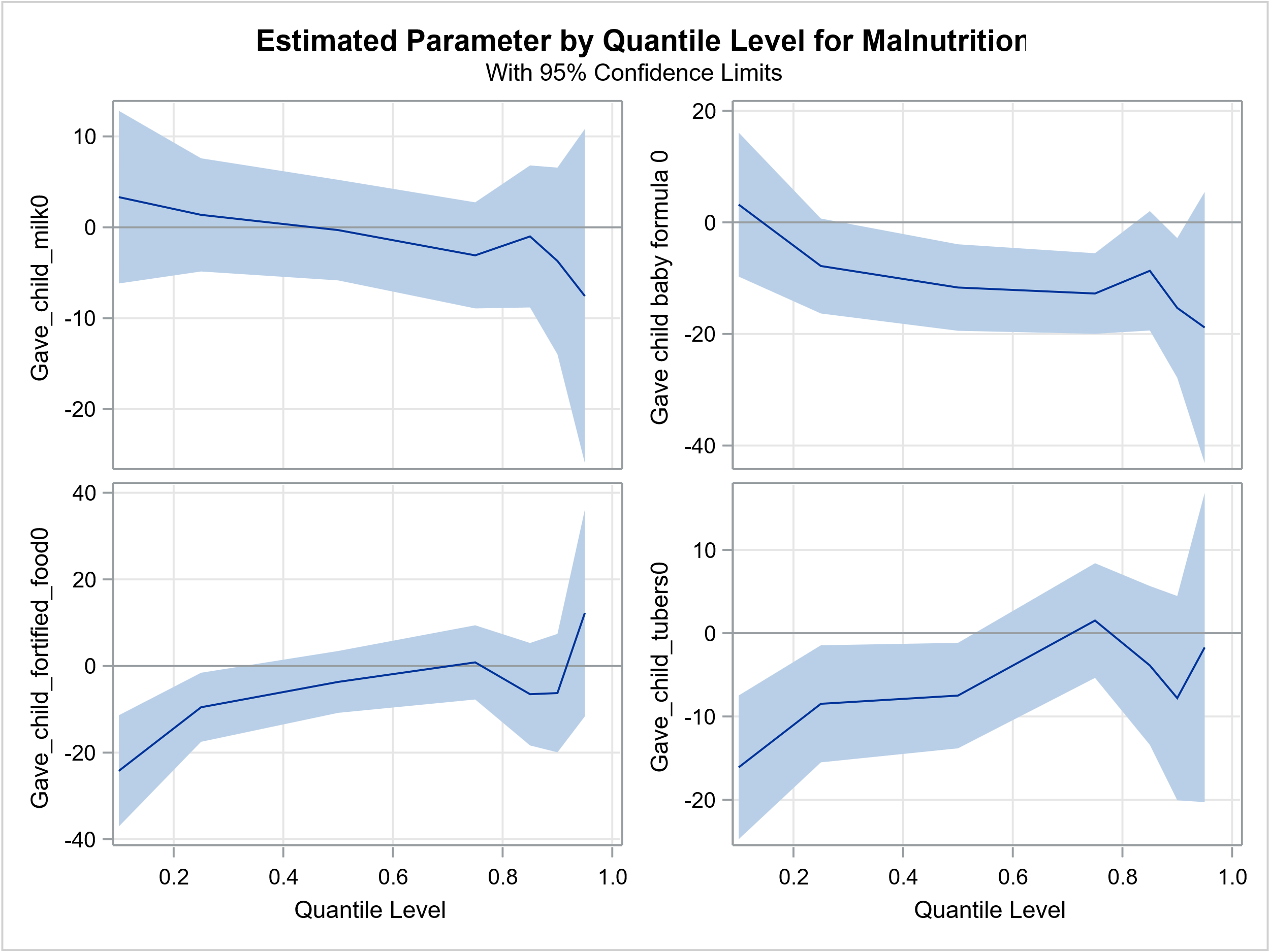


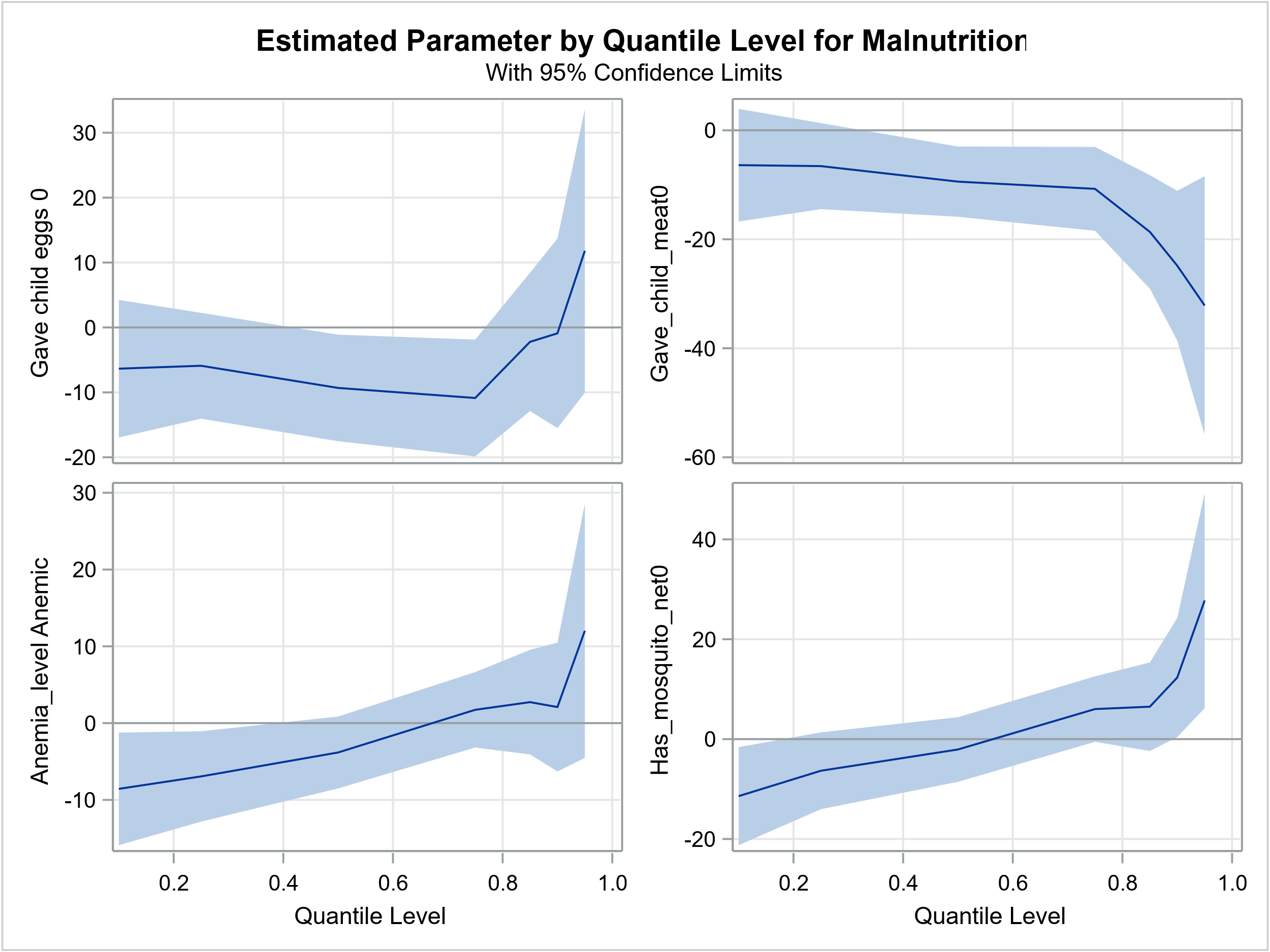


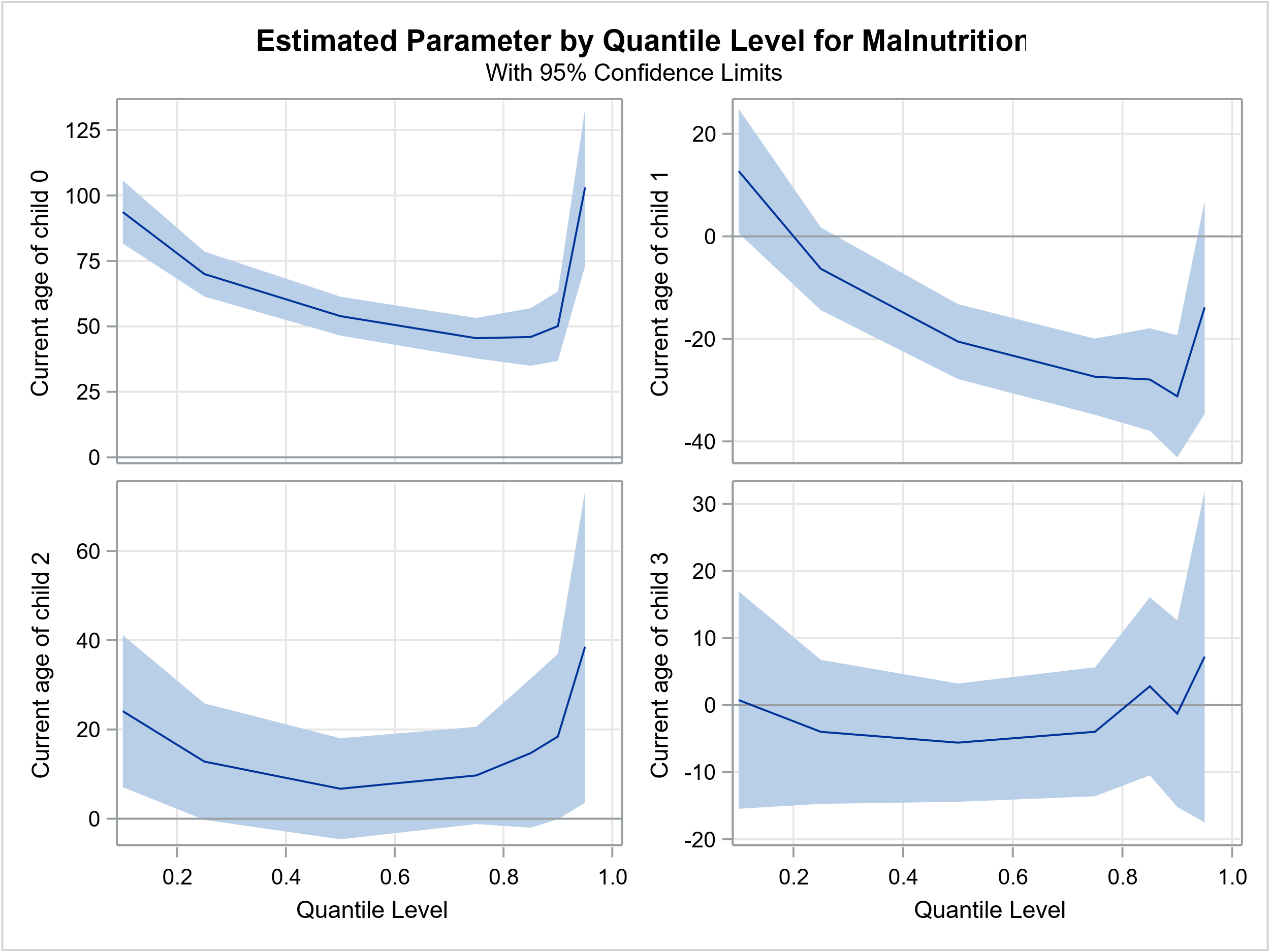


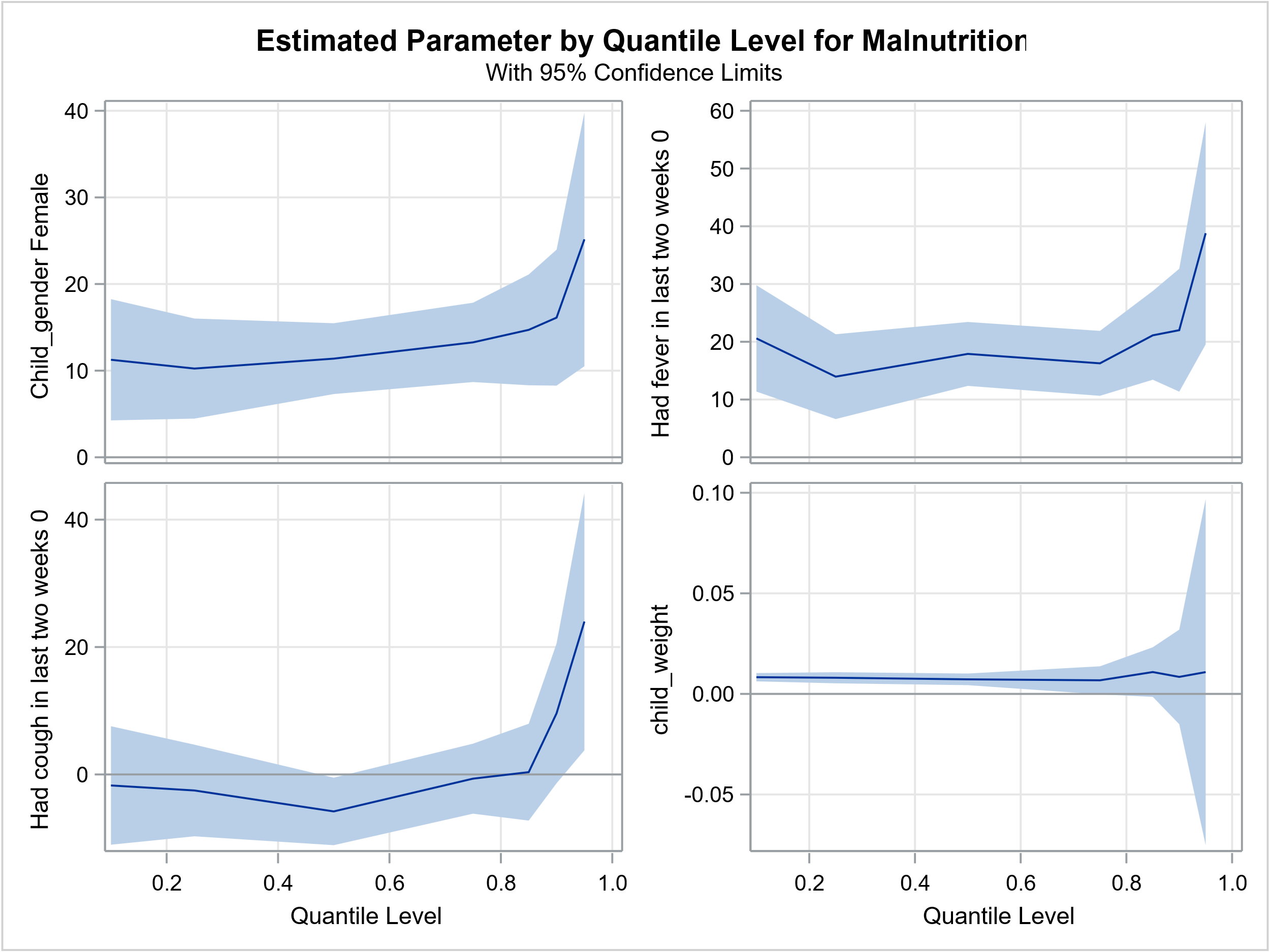


Annexure B

| Abbreviation | Full Term |
| --- | --- |
| DHS | Demographic and Health Surveys |
| HAZ | Height-for-age z score |
| MUAC | Mid-upper arm circumference |
| MAM | Moderate acute malnutrition |
| SAM | Severe acute malnutrition |
| WHZ | Weight-for-height z-score |
| WHO | World Health Organization |
