# Supplementary material for: Quantile regression application to identify key determinants of malnutrition in five West African countries of Gabon, Gambia, Liberia, Mauritania, and Nigeria
Source: Front Public Health. 2025 May 26;13:1520191. doi: 10.3389/fpubh.2025.1520191 (PMC12146170; doi:10.3389/fpubh.2025.1520191)
Supplement: Supplementary file 2 [file Supplementary_file_2.docx]

# Supplementary Table 1 : Parameter estimates at different quantile levels
